# Automatic Segmentation of Monofilament Testing Sites in Plantar Images for Diabetic Foot Management

**DOI:** 10.3390/bioengineering9030086

**Published:** 2022-02-22

**Authors:** Tatiana Costa, Luis Coelho, Manuel F. Silva

**Affiliations:** Instituto Superior de Engenharia do Porto, 1161257 Porto, Portugal; 1161257@isep.ipp.pt (T.C.); mss@isep.ipp.pt (M.F.S.)

**Keywords:** Semmes–Weinstein, monofilament, diabetic foot, automatic

## Abstract

Diabetic peripheral neuropathy is a major complication of diabetes mellitus, and it is the leading cause of foot ulceration and amputations. The Semmes–Weinstein monofilament examination (SWME) is a widely used, low-cost, evidence-based tool for predicting the prognosis of diabetic foot patients. The examination can be quick, but due to the high prevalence of the disease, many healthcare professionals can be assigned to this task several days per month. In an ongoing project, it is our objective to minimize the intervention of humans in the SWME by using an automated testing system relying on computer vision. In this paper we present the project’s first part, constituting a system for automatically identifying the SWME testing sites from digital images. For this, we have created a database of plantar images and developed a segmentation system, based on image processing and deep learning—both of which are novelties. From the 9 testing sites, the system was able to correctly identify most 8 in more than 80% of the images, and 3 of the testing sites were correctly identified in more than 97.8% of the images.

## 1. Introduction

### 1.1. Diabetes and the Risk of Lower-Extremity Amputation

Diabetes is a metabolic disease characterized by uncontrolled blood glucose regulation mechanisms. This chronic disease occurs when the pancreas does not produce enough insulin or when the body cannot use it effectively. In 2014, 8.5% of adults globally, over the age of 18 years, had diabetes. The disease is on the rise worldwide, with the global prevalence in adults being 8.8% of the world population in 2017, with the anticipation of a further increase to 9.9% by 2045. In 2019, diabetes was the direct cause of 1.5 million deaths, with 48% of all diabetes deaths occurring before the age of 70 [1]. Chronic hyperglycemia associated with uncontrolled diabetes damages various organs and systems, causing chronic diabetic complications, leading to disabilities, poor quality of life, and, ultimately, death.

Diabetic peripheral neuropathy (DFN) is a major complication of diabetes mellitus, being the leading cause of foot ulceration and lower-extremity amputations [2]. According to the World Health Organization, lower-extremity amputations (LEAs) are 10 times more common in people with diabetes than in persons who do not have diabetes. According to the Global Burden of Diseases, Injuries, and Risk Factors study, in 2016, about 131 million people (1.8% of the global population) had diabetes-related lower-extremity complications, including 6.8 million amputations [3]. These conditions are common, difficult to treat, and costly, thus requiring proactive preventative assessments by general practitioners and specialists. All patients with diabetes must have their feet evaluated at least once per year for the presence of predisposing factors for ulceration and amputation (neuropathy, vascular disease, and deformities) [4].

### 1.2. The Semmes–Weinstein Monofilament Examination

In diabetic foot management, screening and evaluation are vital for early identification of problems, allowing better patient selection for quick intervention and follow-up. The Semmes–Weinstein 10 gf monofilament examination (SWME) can provide disease-oriented evidence, and it is a consensual tool for predicting the prognosis of diabetic foot patients [5]. It is a non-invasive, low-cost, quick, and easy procedure, often performed in routine evaluations.

The monofilament is a device composed by a nylon filament that is attached to a handle (that can have different shapes). For DFN evaluation, the monofilament has a fixed diameter, and it is calibrated to buckle when a force of 10 gf is applied (though several other values are available when more precise diagnostics are desired). There is some open debate concerning the calibration of the monofilament and the quality of the obtained measures [6], but SWME is undoubtedly a crucial tool for the DFN assessment.

**Figure 1:**
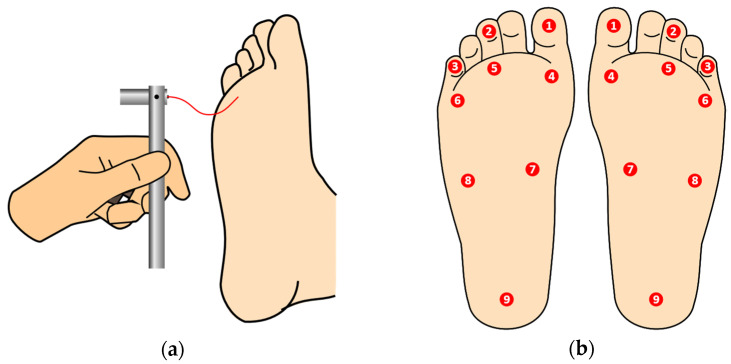
Brief graphical description of the SWME. On the left, (**a**) the application of the monofilament on the plantar area until it starts to buckle, and on the right, (**b**) the plantar testing sites according to international guidelines, on the left and right feet.

For performing the examination, the patient should be comfortable, lying down, facing up, without socks and shoes, so that the plantar area is clearly visible to the examiner. The patient must be informed that the monofilament will touch their feet and they should give feedback as to whether they are feeling the applied pressure or not. Since correct sensitive feedback is crucial for the accuracy of the test, to minimize induced false positives, the patient should not be informed of the beginning of the procedure, and it must be positioned so that it cannot observe the process. The device is positioned and advances perpendicularly towards the skin surface, touching with no impact, while pressure is applied until the monofilament slightly buckles, as depicted in Figure 1a. It should be held in place for approximately 1 s and then released, waiting for the patient’s feedback. This is repeated for all predetermined testing sites, in the left and right foot, as shown in Figure 1b [7]. (The filament should not be used in ulcer sites, callus, scar, or necrotic tissue). The inability of the patient to detect pressure at one or more anatomic sites on the plantar surface of the foot can point to loss of large-fiber nerve function and is a matter for deeper diagnosis or treatment.

The SWME should not require more than three minutes of attention by the examiner, but usually it requires more, and if one considers the time dedicated by other professionals indirectly involved, then the total duration of an entire examination is significant. Due to the high prevalence of the disease, healthcare professionals can be dedicated to this task several working days per month. This a tedious, repetitive task, where human intervention can be minimized, with clinicians’ time being optimized.

### 1.3. Automated and Semi/Automated Evaluation of DFN

After searching major scientific articles repositories, using combinations of keywords, such as “automatic”, “robotic”, “Semmes–Weinstein”, “monofilament”, “plantar”, “diabetes”, and “segmentation”, very few articles were found covering the automation, total or partial, of the SWME, making this a barely explored topic. The most similar project [8] used a mechanical system that was able to perform the monofilament examination in three test sites. The operation was semi-automatic and no closed feedback loops were described, either for monofilament positioning or for patient sensing. The system was evaluated against human performed examinations and, from the four involved patients, only once was a non-agreement case detected. A more complete system is described in [9], and according with the authors description, the foot to be examined had to be pressed against a glass surface, perforated in small, evenly spaced locations. Then, a flatbed scanner acquires the plantar image across the glass. Using an algorithmic approach, five testing sites were segmented, and within these, the position of the nearest hole in the glass was identified. Despite not having been developed, the authors planned to use a robotic arm to apply the monofilament through the holes in the glass surface. Information about the number of acquired images, the evaluation methodology or performance indicators was not provided. The described system raises some hygiene concerns, since the foot must be in contact with the glass surface, and raises some doubts concerning the quality of the monofilament touching sensitivity feedback, since the foot is already pressed against a surface and the monofilament will touch the foot through holes in this surface.

Concerning plantar image segmentation, most of the literature is directed to thermographic images. Despite this distinct image type, many of the reported methodologies can be extrapolated to other image-based approaches. Background segmentation, in particular, can be challenging in thermal images since patients can have their feet or some parts of the feet at near room temperatures, the radiation from the ankles and legs can be difficult to hide during image acquisition, and thermal images do not often provide a clear temperature boundary across different areas [10]. Histogram-shape-based methods, clustering-based methods, entropy-based methods, object-attribute-based methods, and complex genetic algorithms were tested for this purpose with no success in [11]. Image color boosting followed by the active contours without edges (Chan–Vese algorithm) showed to lead to the best results. Pressure footprints, either static or dynamic, can also be a source of plantar-shaped images, and interesting image segmentation approaches, such as in [12,13,14]. However, due to the nature of such images, background segmentation is not required, and the anatomical regions of interest are often quite different from the scope of this article.

In [15], we can find an approach for background segmentation of plantar photographic images. The processing pipeline follows a sequence of image smoothing, Canny edge detection, and a dilation operation, to obtain a rough estimate of the foot contour. To validate their proposal (plantar arch estimation based on a parabolic model), the authors have collected a database of 80 plantar images where the feet were pressed against a transparent surface.

The anatomical structure of the foot is well known, and it can be a useful information source for the segmentation task. In [16], the authors propose an adaptive foot image segmentation algorithm based on morphological partition. The parametric model, developed from anatomical Chinese foot patterns, considers three distinct plantar areas, and achieves very good performance. The GrabCut algorithm is used at a first stage to segment the background from the plantar region of interest. The foot anatomical structure was also used in [17] to support the detection of deformities from angles and dimensional analysis, using image processing. Background segmentation and plantar image binarization was also performed; however, because images were acquired using a specific device with controlled light conditions, the authors were able to use a fixed threshold value.

Another approach, the application of machine learning techniques to plantar images, is proposed in [18]. The authors compare full convolution networks, SegNet and U-Net configurations, on annotated thermal images and test them against a shape-based approach. SegNet provided the best results when using a database of 248 distinct foot images (from 25 healthy controls plus 36 diabetic patients). Machine learning is also used for the segmentation of hallux but the target are x-ray images [19].

Photographic images were used for diabetic foot assessment using soleSCAN, a mobile application [20]. The proposed approach used a set of Gabor filters to identify calluses and to detect pressure “hot spots” in the shoe insole so that they can be relieved in a timely manner. The authors do not make any reference to the SWME.

From the reviewed literature, the authors were not able to find publicly available high-resolution plantar images databases and no established procedures or algorithms exist for the segmentation of the full nine testing sites considered in the SWME.

### 1.4. Manuscript Organization

In an ongoing project, it is the authors’ objective to minimize the intervention of humans in the SWME by using computer vision, and to find the location of the testing sites, combined with an automated testing system, for applying the monofilament plantar sensitivity test to the patient.

In this paper, we present the first part of this project, which consists of a system for automatically identifying the SWME testing sites from digital photographic images. The description of the system as well as the related resources are presented in the following section. We start by providing the details about the development of the plantar images database that has been specifically created for this work and which represents a novelty. Next, the processing system’s overview is shown and the next subsections describe a new approach for the segmentation of the testing sites using image processing techniques, covering background segmentation, hallux and toes segmentation, and plantar sites segmentation. The following section is devoted to the evaluation, results, and related discussion. Finally, we draw the main conclusions and provide ideas for future work.

## 2. Photographic Plantar Image Segmentation for SWME

### 2.1. Plantar Photographic Images Database

The development of the proposed system required a database of plantar photographic images to support laboratory prototyping and testing before deployment. The collection of the database allowed us to gain a priori insight about the effects of light on the plantar surface and to study the variations in foot anatomy—inter and intra gender. The dataset also represents a reference for testing and comparing the performance of different algorithms.

**Figure 2:**
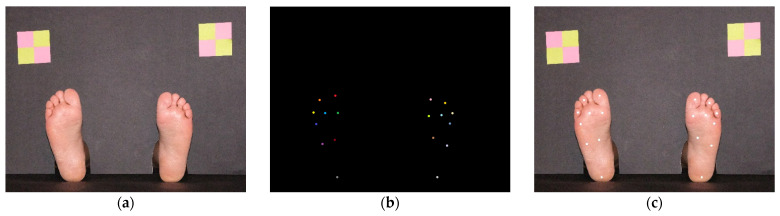
Image taken from the collected database, showing a female foot example. (**a**) Original image; (**b**) mask with 9 testing sites for each foot, manually annotated with different colors; (**c**) original image with superimposed testing sites mask painted in white.

For image acquisition, a simple photographic scenario for placing the feet was prepared. It consisted of two black pieces of paperboard, one for background and another for floor, arranged in a L-shaped setup. The background cardboard had two openings to allow it to be positioned in the ankle area, as seen on Figure 2a. It also had colorful targets, created with yellow and pink sticky notes, with known dimensions, showed on the top left and right corners of Figure 2a. This setup served three purposes: (a) It creates a scenario that is common to all images while hiding all elements that can bring added processing complexity; (b) Easily identifiable image elements with known physical dimensions are visible, which allows us to relate pixel distances with real-world distances; (c) It creates a vision barrier that prevents the patients from observing any procedure, minimizing induced false feedback during SWME. The sticky notes markers were a simple solution for the dimension estimation problem (and color calibration). Possibilities such as Aruco markers [21], AprilTags [22], or similar technologies [23] could be useful, but were considered to bring unnecessary complexity.

The images have been collected using a FujiFilm^TM^ X-T30 camera (FujiFilm, Tokyo, Japan) in an uncontrolled light environment at an approximate distance of 1 m from the paperboard background plane, in perpendicular direction (without specific confirmation). Image acquisition was performed on different occasions, in different days and different rooms. The light and camera position were not controlled in detail, to mimic real-world conditions in a clinical environment. Concerning light sources, different colors and directions can be expected, since, for example, during the day, the main illumination source can be a big window on the left and, during the night, light can come from a lamp on the right and behind the paperboard background. Many light sources can be present simultaneously, leading to multiple shadows and reflections. Concerning position, we also wanted to have flexible conditions ensuring independence concerning this factor. The patient can be laid down slightly more to the left or right, or at different distances from the camera, and furniture can also be subject to changes in a work environment with intense activity. A database collected with minimal constraints allows development of more robust systems adapted to cope with real-world environments where humans and machines must cooperate.

Image registration was made with a resolution of 5328 × 4117, 24-bit RGB RAW format, later converted to JPG with 90% compression ratio. No color correction filters were used during image acquisition. Two layers of annotations were manually performed for all images. The first layer is a binary mask where the feet areas are highlighted (marked as binary 1), allowing us to distinguish them from the background (marked as binary 0). The purpose of this annotation is to evaluate the background segmentation task. The second annotation layer was performed by an experienced clinician who, for each image, created a mask with different colors for each testing site, making a total of 9 + 9 = 18 distinct colors, as shown if Figure 2b. This allows us to evaluate the algorithms’ performance for a single specific testing site or, by converting the mask to a single color, to evaluate the overall performance, as in Figure 2c. The database is composed of 90 distinct plantar images (each image represents 2 feet, making a total of 180 feet images), collected from 6 males and 9 females, Caucasians, without diagnosed foot pathology, with age ranging from 25 to 82 years and an average age of 70.3 years. All individuals had a regular foot anatomy (all five toes, no major deformities).

Plantar areas where the skin has any type of change (ulcer, inflammation, pigmentation, etc.) should be subjected to special attention and shall not be candidates for automated testing procedures (with the current known technology). This was the main reason for using only individuals with healthy feet; although, the inclusion of feet with lesions is envisioned for the future.

### 2.2. System Overview

**Figure 3:**
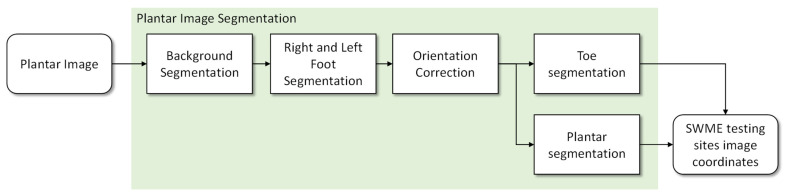
Functional diagram for the proposed plantar image segmentation algorithm.

The proposed segmentation system follows the pipeline represented in Figure 3. All functional blocks are based on formal algorithms, making the pipeline the same, either during the development stage or after deployment. For each plantar image presented at the input, the first step is to separate the background from the feet areas and to find the calibration markers placed on the top left and right of the background plane. After this, each foot area is cropped, with a small outside margin, and its orientation is estimated and realigned to have a vertical orientation. This is an important step since foot orientation varies by more than 50 degrees in the collected images, and the proposed algorithms were developed for vertically aligned feet. Two sub-images are then generated: one containing the toes area and another for the central plantar region and heel. For each of these, algorithms were developed considering their specific anatomic characteristics. All information is finally combined and referenced as coordinates in the original image.

### 2.3. Background Segmentation

As seen in Figure 3, the first step of the proposed system consists of the segmentation of background pixels, distinguishing the skin-covered plantar areas. Skin color segmentation has been previously proposed in several different methods, namely the explicitly defined skin region, non-parametric and parametric skin distribution modeling, and dynamic skin distribution models. The most popular approaches, due to their simplicity, are based on color value thresholding, where the main difficulty is to precisely define skin color boundaries to explicitly establish cutoff ranges. For this, color spaces, such as RGB, HSV, YCbCr, HIS, and YUV, can be explored, and used alone or combined, to achieve optimal results [24,25].

Since the images present bright colored objects (feet) over a dark background, in a uniformed guess we should expect to have a bimodal histogram, making thresholding and segmentation simple. However, due to the unconstrained lighting conditions and the presence of reflections, several bright areas in the background can be observed, despite the dark matte paperboard, making binarization more challenging.

**Figure 4:**
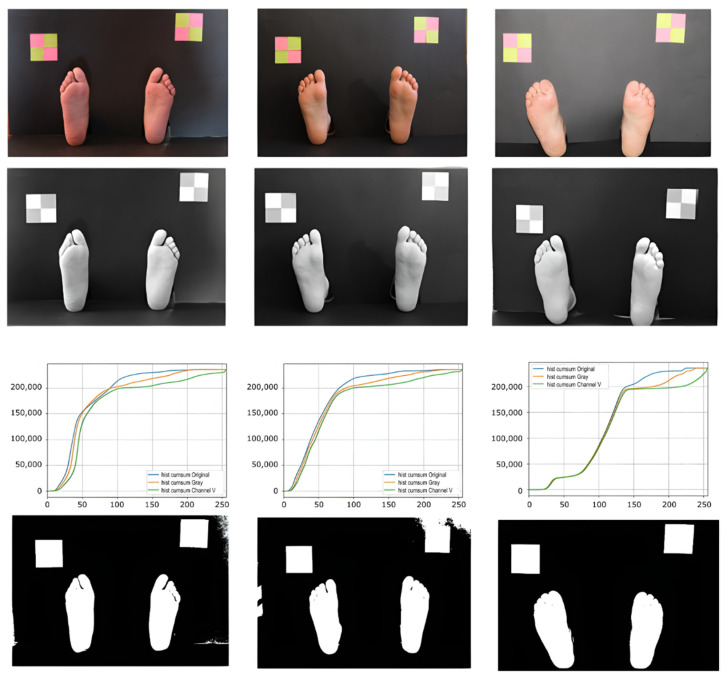
Picture examples along the background segmentation pipeline. First row shows the original images; second row shows the V channel in HSV color space; third row shows the accumulated histograms for the original image (blue), grayscale image (orange), and V channel (green); forth row shows the binarized images.

In the proposed method, we chose to use the V channel of the HSV color space. In this channel, we could observe the intensity of the colors, being possible to differentiate the lighter colors from the darker ones. Some sample images are presented in the first row of Figure 4. In the histogram, a significant peak appears in the darker areas, which correspond to about 90% of the pixels, and in the accumulated histogram, it is possible to observe a very slight slope for the other colors. In Figure 4, third row, we can observe different accumulated histograms based on different color channels. Based on this information, a dynamic threshold value was defined as being two and a half times the peak color value of the channel V histogram. This threshold value was used to binarize the grayscale image obtained from the original. After this, we applied an opening morphological operation to eliminate “lost” pixels. Most of the finally obtained pixel colors fall within the reported skin color ranges.

After localizing the feet areas, a Canny operator was used to find the feet contours. Next, a closing morphological operation was applied to improve the obtained result, followed by a smoothing of the contours.

### 2.4. Hallux Segmentation

**Figure 5:**
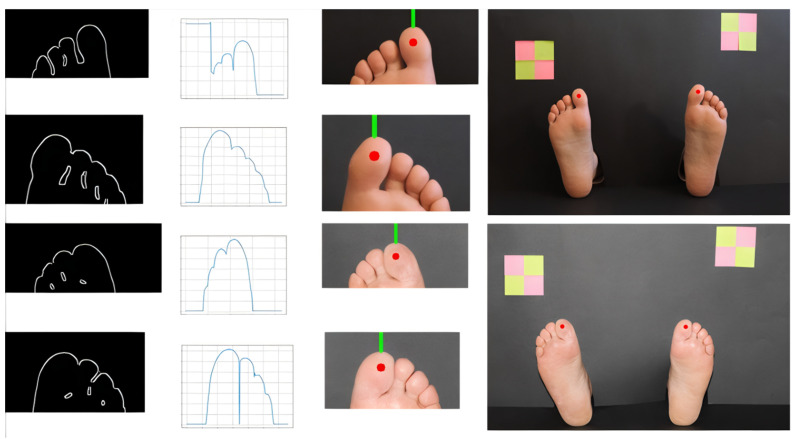
Examples of hallux segmentation and testing site identification. First column shows the foot edges obtained from the background segmentation stage after extraction of the region of interest; second column shows the projected contour values; third column shows the peak contour value and the testing site; forth column shows the testing site remapped on the original image.

Once the areas corresponding to the feet were isolated, the next step consisted of cutting out each of these areas from the original. Threshold cut segmentation techniques were applied in each of these regions, followed by erosion, dilation, and filtering for noise reduction, and segmentation by Canny contour detection. Foot orientation was also estimated by defining a bounding box and calculating the angle of the vertical lines formed by the top and bottom pixel coordinates. The image was then rotated to obtain a vertical representation of the foot. Despite the vertical orientation of the foot, the orientation of the toes, especially those in the extreme left and right positions, can be inward facing, converging to the central axis. Having the result of the contour of the region of interest by the Canny operator, and using anatomy and morphologic knowledge [26] for potential landmarks, a graphical analysis of its behavior in the upper part of the foot was followed, as depicted in Figure 5. To trace the intended curve, it was necessary to traverse each image from top to bottom and from left to right, to analyze the color intensity of each pixel until a transition from black to white was detected, where transition represents the moment when the standing contour is reached. In most images, the hallux corresponds to the peak value of the contour function, but in some cases, the toe next to the hallux has a higher prominence, especially in female feet (only one in our database, making it around 1% of all images). To cope with this, we have considered the hallux position to be the first peak when observing the function from left to right, for the left foot, and from right to left, for the right foot. The identification was based on the contour function and the location of the sign inversion of the first derivative function. This location is expected to be around 20% of the foot width, otherwise an offset must be considered due to a significant angle of the phalange. Having the horizontal location of the hallux testing site, we have defined the vertical location to be below the contour line peak at 10% of the total foot length (based on anatomical information [26]). After this analysis, the translation and rotation of the interest region coordinates was remapped to the original reference, from the sub-image (containing only the toe area) to the original image. In Figure 5, we can observe some processing details of the described algorithm.

### 2.5. Segmentation of Third and Fifth Toes Sites

The toes are subjected to many repetitive efforts and, due to the high loads they support, they end up deforming over the years. These changes translate into significant variations from the regular anatomy of the foot, from individual to individual, and therefore making the identification of test points more challenging. Female feet, often due to the use of shoes that are incompatible with the foot morphology, can present severe skeletal deformations.

**Figure 6:**
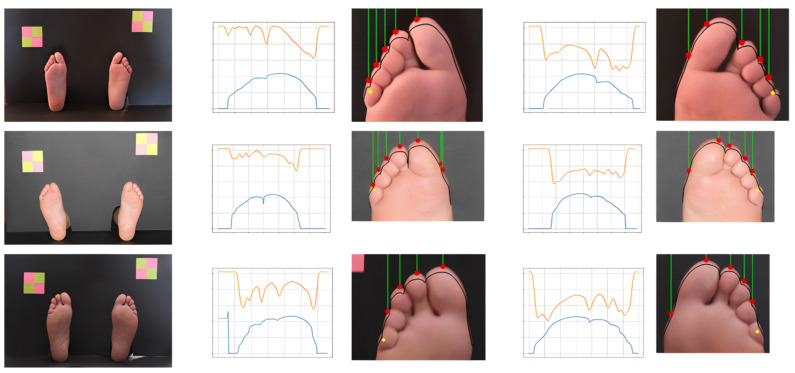
Examples of toe segmentation using a combination of contour and color information, showing left and right feet. The first column shows the original images. In the charts we have the toes’ contour represented in blue and the grayscale pixel intensity along the offset contour represented in orange.

After determining the hallux location (previous stage) we proceed to identity the testing sites located at the third and fifth toes. These are smaller in size, they can be overlapped, and they have a repetitive pattern. Nevertheless, for segmentation purposes, they represent a similar problem, thus we decided to use an approach similar to the one we have previously used for the hallux. These toes are less prominent than the hallux and have similar dimensions and alignments; therefore, they have been treated as an independent group. From the foot image with corrected orientation, segmentation of the area of the toes was performed and the frontal contour of the toes was extracted. Then, the analysis of the obtained curve and its derivative was carried out, each pair of peaks and valleys corresponding to a new toe. Since small toes can be deformed, superimposed, or hidden near neighbor toes, often in female feet, the detection of contour peaks showed to be insufficient. As segmentation strategy, humans rely on morphological landmarks but also on chromatic and brightness information. With this inspiration, we have decided to offset the toe contour vertically, by a distance of 5% in relation to the total foot size, towards the phalange area. This reference track should contain the possible final vertical locations of testing sites. Following this curve, we have extracted the pixel intensity values from the grayscale image. Darker areas represent the inter-toe zone while the brighter areas, between intensity valleys, represent the middle of the toe. Five contour peaks are expected between seven color valleys. The longest valley, corresponding to hallux, should have a length of approximately 40% of the foot width, while the others should have around 15% each. A testing location is marked if it occurs within the expected proportion and if at least one of the color or contour markers are found. In Figure 6, some processing details of the described steps are depicted, showing examples of clearly defined toes (according with the defined rules) and others that were more challenging. By analyzing the location of the darker areas in the original and offset contours we can also estimate how tilted the toes are or how they are morphologically positioned.

**Figure 7:**
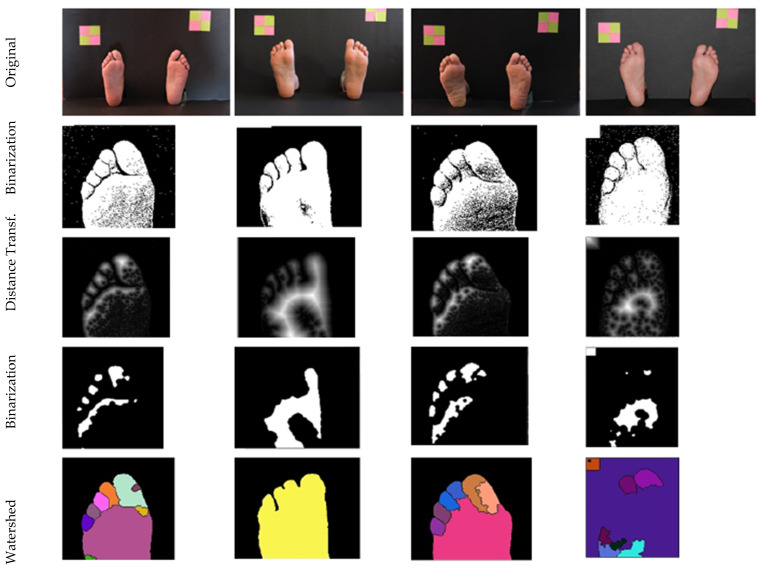
Examples of approaches to toes’ segmentation. (Tested but not used, presented for reference).

Other techniques were also explored and some of the obtained results are presented in Figure 7, for reference. The watershed-algorithm-based approach was the most promising, since it was able to isolate each toe, simplifying posterior processing. However, parameters are sensitive and required careful adjustment.

### 2.6. Segmentation of Heel, Central, and Metatarsophalangeal Sites

Unlike toes, which may be deformed due to the high loads they support, the bone structures that constitute the rest of the foot must guarantee postural stability, having less mobility, being structurally more stable, and having less anatomical variations. Thus, for the segmentation of the test points of the heel, central, and metatarsal regions, it is possible to consider the known morphology of the foot to guide the process.

**Figure 8:**
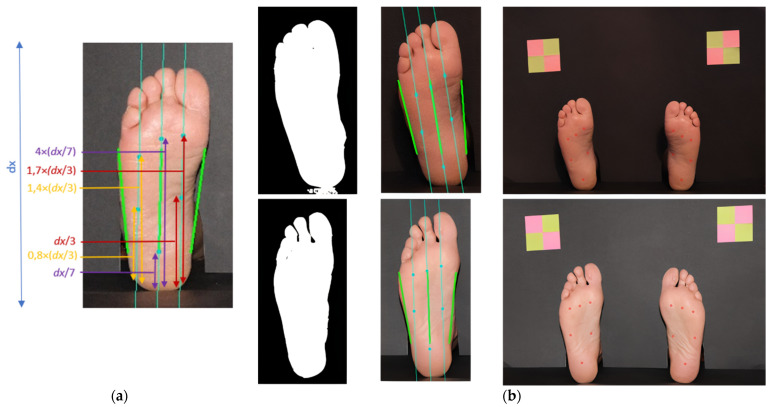
Processing details for the segmentation of the heel, central, and metatarsophalangeal sites. In (**a**), several proportion ratios that were used to draw reference lines can be observed. In (**b**), the application of the proposed algorithm is presented: the first column shows the binary image; the second column shows the left and right border guidelines, as well as the central reference directions for the testing sites; the third column shows the testing sites coordinates remapped on the original image.

Foot orientation was estimated before and, in this case, it is combined with the major left and right border lines, as visible in Figure 8a. By traversing the foot with two imaginary horizontal lines, and by detection of pixel intensity transition, obtained by analyzing the binary image, we were able to find the main foot guidelines. The upper imaginary line was drawn at 40% *dx* (*dx* being the image height) and the lower one at 80% *dx*. Once the four extreme points have been obtained, it is possible to determine the center of the foot and, consequently, the desired orientation, represented in green in the center of Figure 8a. From these it is possible to estimate the position of four additional points located between the central line and each of the side guidelines. We have decided to divide the foot section in four segments because the hallux is proportionally bigger and the fourth and fifth toes are very close together. Hence, with *w* being the foot width, at a given *dx*, the central line is calculated to be at *w*/2. For the other guidelines, marked in light blue in Figure 8a, the leftmost one is located at *w*/4 and the rightmost one is located at 3/4*w*. In the second column of Figure 8b, we can observe other examples for this. The locations of the testing sites within the inner guidelines were defined as a proportion of the foot length, using anatomical landmarks and well-described relative dimensions.

The implementation of the procedure was performed according to the following description. For the task of orientation and identification of plantar points, it was necessary to perform the extraction of the area of each foot and subsequent binarization. From the binarized image of the foot, a row of the image matrix was extracted at 40% of its height. Once these values were obtained, the absolute value of the derivative was calculated to detect the two-pixel intensity transitions—the values that delimit the width of the foot. In addition, the midpoint between the two extremes was also determined to identify the foot axis. Then, the same analysis was performed for a matrix row at 80% of the image, to find the two additional side endpoints, as well as its center. After obtaining the six orientation points, three straight reference lines were traced, corresponding to the two lateral ends and to the center of the foot. Through the straight segment found in the center, it is possible to collect all the necessary data to determine the foot’s orientation angle, as well as the repeated equation of the straight line. (This orientation is different from the one previously calculated, based on a bounding box around the foot).

As previously described, two other points were determined on the upper horizontal guideline. Once these two points were obtained, and based on the slope of foot axis, it was possible to draw two new lines that were used as references for identifying the testing sites points. For this, each line was projected on the binary image of the corresponding foot, in order to detect a new transition of pixel intensities from the bottom to the top. When the transition point is detected, the start of the heel is known. By moving height values on each straight line, it was possible to estimate the points of interest.

To mark the points, the displacement on the lines associated with each one was determined from anatomical knowledge, and the best results obtained were estimated from proportions created based on the height of the areas of each contour, as shown in Figure 8a.

## 3. Results

### 3.1. Background Segmentation

Regarding the background segmentation task, the proposed algorithm was shown to perform very well in most cases. At first, we have evaluated the ability of the algorithm to identify two distinct areas corresponding to the feet, independently of contour quality. Hence, the feet detection accuracy was defined as:

Acc._FD_ = #correctly identified feet blobs/#feet, (1)

Of the ninety images analyzed, only in one case was it found that the segmentation algorithm was not able to correctly separate the background from the existent image elements, and it was not possible to correctly identify the two plantar sub-images. If we consider each foot independently, out of 180 feet considered, a total of 179 were correctly detected and segmented, thus presenting an accuracy rate of 99.4% in foot detection.

Additionally, the edge accuracy was also calculated, having manually created masks as reference. For this evaluation, we have calculated the Hamming weight from the binary XOR between the estimated contour and the human annotated contour. To obtain a resolution invariant dissimilarity metric, we have normalized the result by the total number of image pixels. For two images, *A* and *B*, with identical width, *M,* and height, *N*, composed by pixels *a_xy_* and *b_xy_*, we can define this metric as:

(2) $$d\left(A,B\right)=\frac{1}{M.N}\overset{M}{\underset{x}{\sum }}\overset{N}{\underset{y}{\sum }}\left(a_{xy}⊕b_{xy}\right)$$

Since the output is normalized, we were able to define an error margin, which, in our case, was set at 0.05. Thus, using the images from the previous stage where the feet were correctly identified, considering a 5% error rate, the algorithm was able to correctly identify 97.2% of the foot contours/edges. Hu et al.’s moments, and others’ distances [27,28], were also considered as figures of merit during development, to quantify algorithm’s performance improvement, but they are not presented here since they are image dependent.

### 3.2. Testing Sites Segmentation

The sample that was used to perform the tests for the developed algorithm consists of ninety images of fifteen different people. From these, nine females are distinguished, representing a total of forty-three plantar images, and six males, representing a total of forty-seven plantar images. Different images were collected from each person, thus allowing the study of the behavior of factors such as feet orientation, light incidence, and the distance between objects of known dimensions and the feet. For evaluation purposes, we considered a 5 mm radius around the manually annotated references for the toes, and a 10 mm radius for the central and heel testing site locations (TSL). For *N* images, *i*, an admissible error radius, *r*, and testing site coordinates, $TS_{k}$, and their estimates, $\hat{TS_{k}}$, we can define the location accuracy (*Acc*) as:

(3) $$Acc._{TSL}\left(r\right)=\frac{1}{N}\sum_{i}^{N}{\left[d\left(TS_{k},\hat{TS_{k}}\right)<r\right]}_{i}$$

In Table 1, we can observe the overall results for the full segmentation task, where each row represents a testing site as defined in Figure 1b. Testing site #2, corresponding to the middle finger, showed to be the most challenging to correctly identify, while testing sites #1 and #7 were correctly identified with the highest performance.

Still in Table 1, in the last two columns (entitled Gender), we can observe the results when making a distinction between the male and female feet. For these results, we have considered ninety-four values for each point regarding the male foot, and eighty-six values for each point regarding the female foot. Testing sites #1, #7, #8, and #9 were correctly identified in all cases, while test site #2 had the worst performance. Gender wise, by analyzing the tested samples, it is possible to see that better results were obtained for the male feet than for the female ones. In addition to the added difficulty due to the existence of anatomical deformations that hinder the proper functioning of the proposed strategies, there is also a set of images where the position of the feet or the camera result in more challenging processing conditions.

Another view and analysis of the obtained results consisted of dividing the sample between the right foot and the left foot, as can be seen, respectively, in the central columns of Table 1. From the analysis of the results, it is possible to state that a similar result was obtained, both in the analysis of the right foot and in the analysis of the left foot.

To better evaluate the proposed algorithms and to promote comparisons with other approaches, we have calculated additional performance metrics.

To evaluate the similarity of two binary images, the Jaccard index (as well as the related Sorensen–Dice coefficient) is a robust estimator and a popular choice [29]. This index (also known as Jaccard similarity coefficient or intersection over union (IOU)) is defined for two sets, *A* and *B*, as the size of the intersection set divided by the size of the union set, as follows:

(4) $$J\left(A,B\right)=\frac{\left|A\cap B\right|}{\left|A\cup B\right|}=\frac{\left|A\cap B\right|}{\left|A\right|+\left|B\right|-\left|A\cap B\right|}$$

**Figure 9:**
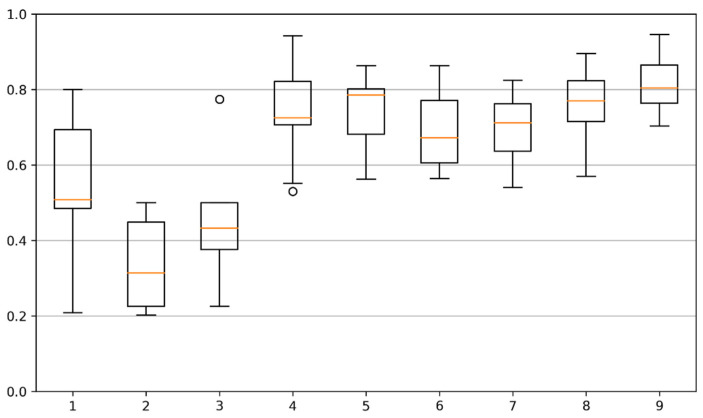
Jaccard similarity coefficient distribution for each testing site (1–9). The orange line represents the median value, dividing the central rectangle in the 25th percentile (below) and 75th percentile (above). The bottom and top lines represent the distribution minimum and maximum, respectively. (Outlier values are also represented as circles, above and/or below the extreme limits.).

In computational terms, since our location sites masks can be represented as bidimensional binary sets, we can use a matrix bitwise operation, being the intersection calculated using the bitwise AND operator. In Figure 9, we can observe a set of box plots with the distributions of the Jaccard similarity coefficient (JSC). For each toe, and for each image, we have calculated JSC between the estimated testing site and the manually annotated mask, when the testing site was present/estimated. We can observe that the scores for the first three testing sites are lower than the other sites, and that the first testing site presents a better result. We can also observe that there is a wider variation of JSC for the first testing site, probably due to a higher morphological diversity. The remaining testing sites have better scores, ranging between 0.7 and 0.8, approximately. These values point to a good overlap between estimated and reference locations.

### 3.3. Time Analysis

The presented algorithms are intended to be integrated into an automatic evaluation system, where a camera will acquire images in real-time. Processing time is therefore an important characteristic to evaluate, in particular, considering that the analyzed images are of very high resolution and that many of the processing operations are computationally demanding. To determine the required time to identify the nine points of interest, we have applied the proposed algorithms to each of the images in the database. The processing times were recorded and stored in a data vector. Each code script was executed three times to overcome possible delays due to the operating system, allowing us to reach a more robust indicator. Subsequently, the average of all the recorded values was performed. Thus, the average time to identify the 9 points of interest in a plantar image was 2.035 s, with a standard deviation of 0.037—the maximum value recorded was 2.078 s and the minimum value was 2.007 s.

The system was developed and tested on a 2.0 GHz 8th generation Intel i7 laptop with 8 Gb of RAM and no dedicated graphics card. The algorithms were implemented in Python and OpenCV, over Windows 10 operating system. Without changing the algorithms, the observed processing times can be improved by using a better hardware setup or by decreasing resolution and color depth. Using images at half the resolution would mean no loss of quality and would translate into a 4-fold decrease in the number of pixels. Using 8 bits of color, instead of 24 bits, would also reduce the image size by 3 times. With these simple changes the same algorithms can run 12x faster, making this enough for a near real time estimation of the testing sites. Using increased step in processing cycles could also improve the algorithm’s performance.

## 4. Discussion

**Figure 10:**
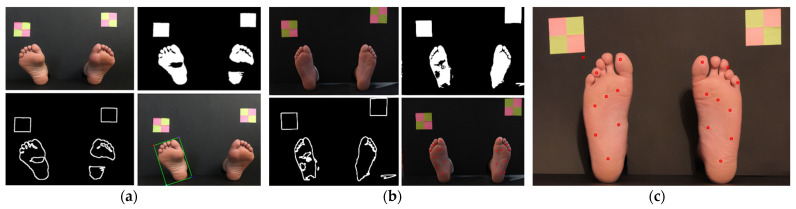
Examples of factors that affect performance: (**a**) feet inclined toward the camera; (**b**) different illumination conditions for the left and right feet; (**c**) dimensional reference marker interference in long feet.

Based on the analysis of the obtained results, it is possible to point out that the algorithm has lower performance when the plantar surface, at the time of image acquisition, is inclined towards the camera—not vertically positioned. This happens since the segmentation of the foot image is conditioned by the substantial increase in the shadow effect, since—with the foot’s inclination—the lighting does not manifest itself uniformly over the plantar region, which leads to parts of the foot having a greater darker shade than desired. Figure 10a illustrates the flaw just described. In the upper left corner is the original image; in the upper right corner is the image after the application of techniques, such as binarization, erosion, and dilation; in the lower left corner is the image as a result of the Canny operator application; and in the lower right corner is the original image with the markings of the detected areas of the feet. To mitigate this, we see two options: (a) have a human assistant for the process that can ensure that the feet pose is aligned with the image capture plane; (b) mount the camera in a mechanical system that can be moved until aligned with the foot plane.

Another factor that negatively influences the performance of the algorithm is the lighting conditions at the time of acquisition of plantar images. Feet morphology and feet pose can lead to shadows and under illuminated areas when light sources are not directed to the plantar area. When there are several light sources with different intensities in the capture environment, the entire image processing is constrained. Consequently, the contour of the foot is not guaranteed in the same way as it would be if there were an even distribution of light incidence. Figure 10b illustrates the described failure, where it is possible to see that the estimated points do not correspond exactly to the desired ones due to the influence of lighting at the time of image acquisition. In the upper left corner is the original image; in the upper right corner is the image after the application of techniques such as binarization, erosion, and dilation; in the lower left corner is the image as a result of the Canny operator application; and in the lower right corner is the original image with the marking of the points of interest identified for the MSW test. The identified problem can be corrected by using dedicated light sources with beam diffusion that can provide uniform illumination conditions, creating a more homogeneously distributed color and reducing shadows.

Furthermore, it was found that in longer feet, the dimensional references, located in the top left and top right, were not in the ideal position. Given the proximity to the foot, when it becomes necessary to perform feet segmentation and extraction of the upper toe contour, it appears that there is an influence of the marker, which ends up being partially highlighted in the image created from the coordinates of the foot identified in the original image. Figure 10c illustrates the described failure, where it is possible to see that the estimated points do not correspond exactly to the desired ones due to the influence of the object of known dimensions. We can observe the estimated and signaled points of interest, where it is possible to see that, under the influence of the object of known dimensions in the binary image, the testing site represented by the number 3 is not within the foot area but located outside. In a final acquisition setup, a bigger background plane can be used as well as smaller markers, positioned in non-intrusive locations.

## 5. Conclusions

The diabetes epidemic is a paramount topic and must be a focus of attention. Limb amputation continues to be one of the main complications associated with the diagnosis of diabetic foot, which highlights the importance of screening. The development of this project is pertinent, as it opened the way for the study and development of a robotic system, whose main purpose is to support the performance of one of the necessary screenings for the timely diagnosis of loss of plantar sensitivity in patients diagnosed with diabetic foot.

In this paper, we have proposed a set of algorithms for the computer vision module, one of the components of the automatic SWME system. The main objective was defined as the identification of the foot testing sites based on a digital photographic image acquired in a real environment, without specific constraints. We started by presenting the current state of the art, by providing a literature revision of the main, previously published articles in related topics. To the authors’ knowledge, no work has been carried out to address this specific problem, and no databases are publicly available to support such developments.

For these purposes, we collected a new database of high-resolution plantar photos, composed of 90 distinct images, from 9 females and 6 males, with varied ages. The COVID-19 pandemic has constrained the image acquisition, but the authors plan to continue this work and make the database available to other researchers.

For the automatic segmentation of the testing sites, we have presented a set of algorithms which have been described in detail. Background segmentation, hallux segmentation, and plantar and heel segmentation are the main processing blocks of the system.

The final pipeline was evaluated in different ways: block-by-block, end-to-end, females vs. males, and left foot vs. right foot. Overall, from the 9 testing sites, the system was able to correctly identify at least 7 in more than 86% of the images, and 4 of the testing sites were correctly identified in more than 98.3% of the images. The identification of the testing sites in small toes proved to be a challenging task, mainly due to anatomy variations, and further research is envisioned.

After presenting the results we have thoroughly discussed the strong and weak points of the processing algorithms. Mitigation suggestions were provided for the later.

As a conclusion, we find that the proposed system performed very well in the testing conditions and the problems were clearly identified. Improvements are foreseen and some suggestions for future developments are presented in the next section.

We would also like to add that some of the constraints encountered during the development of this project, due to the COVID-19 pandemic, reinforced the importance of the proposed system. The evaluation of the diabetic foot was suspended in some healthcare institutions; therefore, it was not possible to collect images from real patients. The automation of SWME allows the patient to be examined even in the case of respiratory infections, since the objective is to minimize human intervention.

## 6. Future Work

The challenging conditions that have been encountered motivate the authors to carry out additional developments in the future. The segmentation of small toes was the module with worst performance indicators, and it will be the main focus of improvement. The use of other approaches, in particular those based on artificial intelligence techniques, is planned. In distinct problems, segmentation tasks have been successfully handled by deep learning networks, as in [30,31]. It is the authors belief that plantar image segmentation can also benefit from such algorithms. Following this approach, due to the high number of involved parameters, it is also planned to continue with plantar image collection to feed the training algorithms and achieve robust models. Images of feet with lesions must also be included in the database and this will represent a new problem, the segmentation of lesions in the plantar area. The new images will require manual annotation by a human expert, which can be a time-consuming task. Another possible development is to create a semi-automatic annotation system where the segmentation algorithm can be used to suggest the annotation marks. Concerning human annotation, it would be also interesting to have multiple annotations from different experts, to estimate the human agreement for each location and to understand the admissible error range for each testing site, which is presumably different for a small toe and the heel, for example.

Since the feet are responsible for supporting the weight of the body, their bone structure is one of the main sources of plantar pressure points. In fact, some of test sites are specially aimed to the location of interphalangeal joints. The estimation of the hidden bone structure from photographic images, such as in [32], could have particular interest to improve the plantar sensitivity test and enforce the importance of the SWME.

Additionally, for integrating our vision system with a robotic manipulator that can perform the SWME, the use of an RGB + depth camera could be an option to consider. The additional data included in the depth channel can highly simplify the background segmentation task and the toes segmentation task, while creating a connection between the robotic manipulator and the human feet in a real-world clinical context.

## Figures and Tables

**Table 1:** Segmentation accuracy for each testing site (TS), calculated as the ratio between correctly identified sites over total sites, for a given site location. All values are presented in percentage (%).

#TS	Overall	Foot		Gender	
		Left	Right	Male	Female
1	99.4	98.9	100.0	100.0	98.8
2	35.6	36.7	34.4	19.2	53.5
3	57.8	62.2	53.3	56.4	59.3
4	88.9	86.7	91.1	96.8	80.2
5	85.9	82.2	88.9	95.7	74.4
6	86.1	83.3	88.9	96.8	74.4
7	99.4	98.9	100.0	100.0	98.8
8	98.3	97.8	98.9	100.0	96.5
9	98.3	97.8	98.9	100.0	96.5

## Data Availability

Data is not publicly available, but the authors can provide limited access for research purpose in the condition of clear authorship attribution.
